# Mindfulness and Other Psycho-Social Resources Protective Against Mental Illness and Suicidality Among Gay Men

**DOI:** 10.3389/fpsyt.2018.00361

**Published:** 2018-08-09

**Authors:** Jen Wang, Michael Häusermann, Anne-Emmanuelle Ambresin

**Affiliations:** ^1^Interdisciplinary Division for Adolescent Health (DISA), Lausanne University Hospital (CHUV), Lausanne, Switzerland; ^2^Dialogai, Geneva, Switzerland

**Keywords:** psycho-social resources, mindfulness, depression, suicidality, victimization, homosexuality

## Abstract

**Background:** There is considerable evidence of health disparities among gay men characterized by higher levels of stress and distress. Psycho-social resources have been linked to numerous positive health outcomes and shown to act as buffers in the stress-distress pathway.

**Methods:** With data from the 3rd Geneva Gay Men's Health Survey carried out in 2011 using time-space sampling (*n* = 428), a relatively elaborate profile of 14 psycho-social resources—including mindfulness—is presented. Using their original scores, latent class analysis created an index variable dividing the respondents into meaningful groups. Psycho-social resources—the index variable as well as each resource individually—were then compared to two recent outcomes—i.e., serious mental illness in the past 4 weeks and short-term disability in the past 2 weeks—using a series of logistic regression models, controlling for all other psycho-social resources and socio-demographic confounders. To assess their potential role as buffers, a similar series of logistic regression models were erected using victimization and three outcomes—i.e., major depression, suicidal ideation, and suicide attempt—in the past 12 months.

**Results:** According to the latent class analyses, (1) 5.1% of this sample had a low level of psycho-social resources (i.e., one standard deviation (SD) below the group means), (2) 25.2% a medium-low level, (3) 47.4% a medium level (i.e., at the group means), and (4) 22.2% a high level of psycho-social resources (i.e., one SD above the group means). Psycho-social resources appeared to strongly protect against recent mental morbidity and buffer against the impact of victimization on major depression and suicidality in the past 12 months, reducing the adjusted odds ratios below statistical significance. The explained variance and the individual psycho-social resources which remained independent in the models differed for each outcome.

**Conclusions:** There may be disparities in several psycho-social resources among gay men, and as strong compensatory and protective factors, they may explain in part the well-established disparities in stress and distress in this population. While multiple psycho-social resources should be promoted in this population, gay men under 25 years should receive particular attention as all three disparities are most pronounced in this age group.

## Introduction

Psycho-social resources have been linked to positive mental and physical health outcomes (1) and even reduced mortality in the general population (2), but since it has been shown that psycho-social resources are not distributed equally (1, 3), they may also help explain health disparities as important mediators and moderators for poor health outcomes (4). For example, mastery has been shown to mediate the relationship between sex (5), education (6), and poor physical health (7, 8) with depressive symptoms.

A review of the literature on stress and health has underscored the role of psycho-social resources such as mastery, self-esteem, and social support as key buffers in the stress-distress pathway (9). For example, longitudinal data from the Canadian National Population Health Survey (NPHS) established a link between mastery and social support with depression symptoms, but mastery was also shown to moderate and mediate the negative impact of stressors such as daily stress on depression symptoms (8). However, the review also noted the adverse coincidence of more acute and chronic stressors and fewer psycho-social resources among groups with lower social status (9).

Victimization constitutes a severe stressor which entails immediate psychological sequelae in both the general population (10) and sexual minorities (11), although research on its long-term effects has been equivocal (10). Victimization is significantly more prevalent among sexual minority adults and teens than their heterosexual counterparts (12–14). In Add Health—a longitudinal study of adolescent health in the US—victimization and same-sex attraction were independently associated with depressive symptoms and suicidality (15), but structural equation modeling with cross-sectional data from the Chicago Youth Risk Behavior Survey (YRBS) showed that victimization constitutes the pathway between sexual identity and suicidality (16).

A considerable international evidence base documents the increased risk of mental morbidity and suicidality among sexual minorities (17). Contributing to this evidence base (18, 19), previous findings from Switzerland have also revealed marked differences in underlying personality traits such as neuroticism which appear to account in part for the increased risk (19). This finding points to the potential relevance of other long-standing factors such as psycho-social resources, yet they remain relatively under-researched among sexual minorities (20).

Dispositional/trait mindfulness has been associated with other psycho-social resources—e.g., self-esteem, empathy, attention, cognitive reactivity, rumination, positive affect, life satisfaction, and vitality—long-standing personality traits—e.g., conscientiousness and neuroticism—as well as mental illness—e.g., depression and anxiety—and to a lesser extent suicidality (21–23). Two population-based studies in Europe found that mindfulness scores for select facets differ by sex, age, education, and income (24, 25), but there have been no comparisons by sexual orientation to date. Only two recent studies have published mindfulness data on sexual minorities: a national online convenience sample of gay men aged 40 years and over in Australia (26, 27) and a national online convenience sample of Latino sexual minority youth aged 14–24 years in the US (28).

In accordance with efforts to conceive (29, 30) and present (31) a more complete view of mental health in a population beyond mental illness alone, we forward a relatively elaborate psycho-social profile (4) of gay men in order to characterize the psycho-social resources—including mindfulness—and their distribution in a probability sample of a stigmatized group with low social status, juxtapose them alongside well-established outcomes of mental illness and suicidality, and assess their potential role as buffers in the stress-distress pathway in a population with higher levels of both stress and distress.

## Methods

The third Geneva Gay Men's Health Survey (GGMHS) was carried out in 2011 with a focus on mental health literacy, mental illness, and suicidality, and the methods have been described in detail elsewhere (32).

### Procedure

Briefly, the target population consisted of gay-identified men and other men who have sex with men who access meeting points—both real and virtual—in Geneva, Switzerland. All three waves employed time-space sampling, a multi-stage randomized sampling scheme developed by the US Centers for Disease Control and Prevention (CDC), involving mapping of meeting points, enumeration of visits, and random selection of both venues and participants (33, 34). Men were informed and invited to participate in a general health survey, and consenting respondents were given a unique access code to complete the anonymous survey in French either immediately at laptops provided on-site or later at a time of their own choosing online. In 2011, 486 gay men participated in the survey (response rate 38%), whereby 428 respondents have complete data for the variables used in this publication.

### Measures

Fourteen psycho-social resources were included in the 2011 survey to yield a more complete picture of mental health and well-being, and they have been organized into 6 families to facilitate reader navigation in the tables.

Core self-evaluation includes 1) mastery and 2) internalized homophobia (aka acceptance of one's homosexuality). Mastery or self-efficacy was measured by the Sense of Mastery Scale (35) which includes 7 items measured on a 4-point scale, with the total score ranging from 7 to 28 (Cronbach's α = 0.79). A key component of self-acceptance among gay men, self-acceptance of one's homosexuality was measured in part by the negatively valenced Internalized Homophobia Scale (IHP-R) (36, 37) which includes 5 items measured on a 5-point scale, with the total averaged score ranging from 1 to 5 (Cronbach's α = 0.85).

Conative resources include 3) purpose in life, 4) hedonism, 5), altruism, 6) religion, and 7) spirituality. Representing a eudaimonic orientation, purpose in life was measured by one question from the life of meaning sub-scale in the Approaches to Happiness Questionnaire—“My life serves a higher purpose (has meaning)” (38)—measured on a 4-point scale. Hedonism was measured by one question representing the basic value hedonism in the Schwartz Value Scale (39)—“He seeks every chance he can to have fun. It is important to him to do things that give him pleasure.”—measured on a 6-point scale. Altruism was measured by one question representing the basic value of benevolence in the Schwartz Value Scale (39)—“It's very important to him to help the people around him. He wants to care for their well-being.”—measured on a 6-point scale. Religion and spirituality were each measured with one question from the Midlife in the US (MIDUS) survey (40)—“How important is [religion/spirituality] in your life?”—on a 4-point scale.

Cognitive resources include several skills targeted by mindfulness training and meditation—i.e., 8) mindful attention (aka acting with awareness), 9) pausing before reacting, and 10) non-rumination. Originally, the 5-item Mindfulness Attention Awareness Scale (MAAS) (41) was chosen to assess mindfulness and 2 items from the Freiburg Mindfulness Inventory (FMI) (42) to assess emotion management. However, reliability testing showed that a 4-item solution for the MAAS had slightly higher Cronbach's α (0.80) than the original 5-item scale, with the 4 core items targeting attention or acting with awareness. The 2 items selected from the FMI to assess emotion management had unacceptable internal consistency (Cronbach's α = 0.49), but one item from the FMI about being easily lost in thoughts and feelings had good reliability with the excluded MAAS item about being preoccupied with the past or future to create an indicator for ordinary rumination (Cronbach's α = 0.69) or rather “non-rumination” in keeping with the indirect assessment approach and positive coding of the MAAS. The single remaining item from the FMI pausing before reacting is kept as an indicator of emotional reactivity. All items were measured on a 6-point scale from the MAAS, yielding an averaged score from 1 to 6.

Affective resources include 11) positive affect and 12) life satisfaction. Positive affect (in the past 4 weeks) was assessed by the Mental Health Index (MHI-5; 5 items, standardized score from 0 to 100, Cronbach's α = 0.83) from the Medical Outcomes Study (MOS) 36-Item Short-Form Health Survey (SF-36) (43). Recommended by the EUROHIS project on harmonizing indicators for health interview surveys in Europe (44), the MHI-5 includes items representing both positive and negative affect but is scored positively. Life satisfaction was measured by a single question from the World Values Survey (45)—“All things considered, how satisfied are you with your life as a whole these days?”—on a 10-point scale.

Often considered a measure of positive mental health, 13) vitality, as measured by the Energy Vitality Index (EVI; 4 items, standardized score from 0 to 100, Cronbach's α = 0.78) from the Medical Outcomes Study (MOS) 36-Item Short-Form Health Survey (SF-36) (43) and recommended by EUROHIS (44), is a psycho-somatic resource.

Social resources include 14) positive relations with others. EUROHIS (44) recommends the 3-item Oslo Social Support (OSS-3) scale (46). Given its poor internal consistency (Cronbach's α = 0.53), however, two additional items on satisfaction with personal relationships and loneliness from the personal relationships facet of the WHOQOL-100 (47) were added to the OSS-3, yielding better internal consistency for a 5-item indicator on positive relations with others (standardized score from 0 to 100, Cronbach's α = 0.68).

Several standard indicators of mental morbidity and suicidality were measured in this survey. The prevalences of these main health outcomes have already been reported (32).

#### Temporary disability

Short-term disability (in the past 2 weeks)—i.e., reducing or stopping usual activities—was measured separately for physical and mental health, using a series of questions recommended by EUROHIS (44).

#### Serious mental illness

Serious mental illness (in the past 4 weeks) was measured using the K6 with 6 items describing negative affect and a cut-off point at 13 on a 0–24 scale (48).

#### Mental illness

Depression and anxiety were assessed by a series of questions recommended by EUROHIS (44)—i.e., 1) self-reported history of chronic depression and anxiety taken from a standardized check-list of chronic conditions (49) modified to yield both 12-month and lifetime prevalences and 2) assessment of 12-month major depression by the WHO Composite International Diagnostic Interview Short Form (CIDI-SF) (50).

#### Suicidality

12-month and lifetime suicidality were measured using Paykel's items covering suicidal ideation (“Have you ever thought of taking your life, even if you would not really do it?”), suicide plans (“Have you ever reached the point where you seriously considered taking your life or perhaps made plans how you would go about doing it?”), and suicide attempt (“Have you ever made an attempt to take your life?”) (51).

#### Victimization

A series of questions from the 1997 Swiss Health Survey (52) was adapted to yield 12-month and lifetime prevalences of verbal violence (insults and threats), physical violence, sexual harassment, rape, and robbery (in and outside the home), and the corresponding results from the 2002 and 2011 Geneva Gay Men's Health Surveys have been published in a local report (53). Since most studies use a global index of victimization, anyone experiencing any of the aforementioned forms of violence in the past 12 months was considered a victim of violence.

### Analysis

Seven of the 14 psycho-social resources involve scales with multiple items which were combined in accordance with their original scoring procedures, yielding scores in different ranges which have been maintained to facilitate comparison of these sample means with others. Their internal consistency was assessed by reliability testing. Improvements on two original scales were made for cognitive and social resources as detailed in the previous section. Pearson correlation coefficients were calculated for each of the 14 psycho-social resources with all the others, using a two-tailed test of significance.

Latent class/profile analysis was carried out to identify latent sub-groups of the respondents, according to their responses for all 14 psycho-social resources. Since there was no theoretically expected number of clusters, we planned an initial run of 1–6 classes given the relatively large number of indicators. The original intent was to account more precisely for different types of ordinal and continuous variables; however, such models had difficulty reaching convergence past 3 classes. By increasing the number of variables treated with Poisson and decreasing the number of variables treated as ordinal, workable models could be erected through 5 classes. Comparing the model fit statistics, a 4-class model was found to yield the lowest Bayes Information Criterion (BIC = 23001.52), together with the most interpretable and meaningful classes.

As no item response probabilities were generated for the continuous variables, analysis of variance (ANOVA) was carried out for each of the 14 psycho-social resources, generalizing the *t*-test for 4 classes with Tukey correction for multiple comparisons. These findings yielded analogous information on response patterns for each class and psycho-social resource, confirming both robust discrimination between classes and meaningful class membership.

In order to assess the relationship between the 4-class index of psycho-social resources and mental illness and suicidality health outcomes, ANOVA with Tukey correction was used once again for the continuous variables, while contingency tables and the chi-squared test were used for dichotomous variables.

Similar analyses with socio-demographic variables—i.e., place of residence, commune size, cohabitation, partnership status, age, nationality, education, employment status, and sexual identity—were carried out for health outcomes and psycho-social resources to identify potential confounders. Unemployed men demonstrated significantly poorer health outcomes and lower levels of psycho-social resources, as did the youngest men (<25 years). Upon closer examination, men under 25 years demonstrated the lowest levels of mindful attention, non-rumination, and positive affect.

A series of binary logistic regression models was erected in order to quantify and elucidate the relationship between psycho-social resources and common mental illness outcomes. In the first series, recent mental illness outcomes—i.e., serious mental illness in the past 4 weeks and short-term disability due to mental/emotional problem in the past 2 weeks—were each taken as dependent variables and compared to current/recent levels of psycho-social resources. Since findings using the 4-class summary index of psycho-social resources were highly significant, similar analyses were performed using all 14 psycho-social resources as independent variables in order to identify the most relevant for each outcome. In Model 1, all 14 psycho-social resources were entered as a single block, thereby yielding adjusted odds ratios (AOR) for each psycho-social resource whilst simultaneously controlling for all the others. In Model 2, all 14 psycho-social resources were entered as a single block, followed by back-step elimination of select socio-demographic covariates—i.e., cohabitation, age, nationality, education, and employment status—thereby yielding AOR for each psycho-social resource whilst simultaneously controlling for all the others and any significant socio-demographic confounders. In Model 3, the most parsimonious logistic regression model was identified by back-step elimination of all 14 psycho-social resources and the aforementioned socio-demographic covariates, thereby yielding AOR for each significant psycho-social resource whilst simultaneously controlling for all other significant psycho-social resources and socio-demographic confounders. (NB: Due to the relatively small number of suicide attempts in the past 12 months, the only socio-demographic covariate included in Models 2–3 for this outcome was age).

Since the latent class solution for current/recent psycho-social resources appeared to distinguish strongly even for mental illness and suicidality outcomes in the past 12 months, we decided to explore potential meditation effects of psycho-social resources in the classic stressor-distress pathway. The stressor was any experience of victimization in the past 12 months, and the 3 distress outcomes were major depression (according to CIDI-SF), suicidal ideation, and suicide attempt in the same timeframe. For each outcome, a second series of 3 logistic regression models was carried out with victimization accompanying the 14 psycho-social resources in the single block in Models 1–2 and constituting the sole block entry variable in Model 3, thereby yielding AOR for victimization, adjusting for psycho-social resources and/or socio-demographic confounders. Data analysis was performed using IBM SPSS Statistics 23 and STATA 15 for PC.

## Results

Table 1 displays the mean scores with standard deviations of each of the 14 psycho-social resources according to the original scales and the simple correlations between them. Higher scores indicate a higher level of all psycho-social resources, except for internalized homophobia where higher scores indicate a lower level of self-acceptance of one's homosexuality. Since the scales for the cognitive and social resources have been altered for this publication, the means and standard deviation (SD) for the original 5-item MAAS (mean 4.23, *SD* 0.93) and the 3-item OSS (mean 9.80, *SD* 2.25) scales are presented for reference here. The mean scores will be compared with available population mean scores in the Discussion section. The correlation matrix shows that most indicators are significantly correlated with each other at weak to moderate levels. Of note, altruism, religion, and spirituality are correlated weakly if at all with other indicators.

**Table 1:** Means, standard deviations, and zero-order correlations of psycho-social resources among gay men in Geneva, Switzerland, in 2011(*n* = 428).

**Family**	**Variable**	**Range**	**Mean**	**SD**	**1**	**2**	**3**	**4**	**5**	**6**	**7**	**8**	**9**	**10**	**11**	**12**	**13**	**14**
**CORE SELF-EVALUATION**																		
1	Mastery	7–28	20.54	3.56	—													
2	Internalized homophobia (aka acceptance of one's homosexuality)	1–5	1.84	0.89	−0.21^***^	—												
**CONATIVE**																		
3	Purpose in life	1–4	3.02	0.78	0.39^***^	−0.11^*^	—											
4	Hedonism	1–6	4.56	1.10	0.15^**^	−0.19^***^	0.18^***^	—										
5	Altruism	1–6	4.45	1.18	0.08	−0.10	0.18^***^	0.25^***^	—									
6	Religion	1–4	1.75	1.03	0.04	0.24^***^	0.20^***^	−0.03	0.08	—								
7	Spirituality	1–4	2.57	1.15	0.05	0.13^**^	0.15^**^	0.05	0.08	0.45^***^	—							
**COGNITIVE**																		
8	Mindful attention (aka acting w/ awareness)	1–6	4.42	0.96	0.39^***^	−0.21^***^	0.18^***^	0.03	0.06	0.07	0.02	—						
9	Pausing before reacting (aka emotional reactivity)	1–6	3.63	1.20	0.48^***^	−0.17^***^	0.21^***^	−0.02	−0.07	−0.01	−0.07	0.55^***^	—					
10	Non-rumination	1–6	4.13	1.18	0.24^***^	−0.003	0.22^***^	0.10^*^	0.10^*^	0.08	0.14^**^	0.16^**^	0.10^*^	—				
**AFFECTIVE**																		
11	Positive affect	0–100	66.10	17.61	0.55^***^	−0.27^***^	0.32^***^	0.14^**^	0.05	−0.04	0.02	0.43^***^	0.51^***^	0.19^***^	—			
12	Life satisfaction	1–10	7.24	1.95	0.53^***^	−0.35^***^	0.41^***^	0.18^***^	0.02	0.06	0.02	0.36^***^	0.39^***^	0.14^**^	0.59^***^	—		
**PSYCHO-SOMATIC**																		
13	Vitality	0–100	58.63	17.09	0.46^***^	−0.19^***^	0.32^***^	0.20^***^	0.02	−0.01	0.02	0.34^***^	0.40^***^	0.18^***^	0.66^***^	0.50^***^	—	
**SOCIAL**																		
14	Positive relations with others	0–100	64.29	18.92	0.52^***^	−0.24^***^	0.38^***^	0.16^**^	0.11^*^	0.05	−0.03	0.28^***^	0.38^***^	0.19^***^	0.52^***^	0.60^***^	0.43^***^	—

* *p < 0.05*,

** *p < 0.01*,

*** *p < 0.001*.

Since most of these indicators do not have cut-offs to facilitate interpretation, the respondents were grouped according to their scores for all 14 psycho-social resources into 4 distinct classes using latent class analyses: 1) 5.1% of this sample had a low level of psycho-social resources, 2) 25.2% a medium-low level, 3) 47.4% a medium level, and 4) 22.2% a high level of psycho-social resources.

In lieu of a standard item probability graph, Table 2 presents the statistical comparison of mean scores for all 14 psycho-social resources by latent class. The respondents in each class differ significantly from all others along 7 indicators. The respondents' responses to altruism, religion, and spirituality do not distinguish between these classes at all. The means of the class with the medium level, which include nearly half the respondents, correspond neatly to the mean scores for the overall group for each indicator, making it a true intermediate group. For the class with a low level of psycho-social resources, most of their mean scores are more than one standard deviation below the medium (i.e., overall) group mean, whereas for the class with a high level of psycho-social resources, many of their mean scores are more than one standard deviation above the medium (i.e., overall) group mean.

**Table 2:** Latent class model as characterized by psycho-social resources among gay men in Geneva, Switzerland, in 2011 (*n* = 428).

					**Psychosocial resources**				
					**Low (L)**	**Medium-low (LM)**	**Medium (M)**	**High (H)**	
**Family**	**Variable**	**Range**	**Mean**	**SD**	**Mean (SD)**	**Mean (SD)**	**Mean (SD)**	**Mean (SD)**	**Statistical significance**
**CORE SELF-EVALUATION**									
1	Mastery	7–28	20.5	3.58	15.3 (3.92)	18.4 (3.13)	20.7 (2.68)	23.7 (2.58)	L < LM < M < H^***^
2	Internalized homophobia (aka acceptance of one's homosexuality)	1–5	1.84	0.90	2.50 (0.96)	2.15 (1.08)	1.75 (0.80)	1.54 (0.67)	L LM > M H^*^
**CONATIVE**									
3	Purpose in life	1–4	3.01	0.79	2.41 (0.67)	2.68 (0.85)	3.05 (0.74)	3.43 (0.58)	L LM < M < H^**^
4	Hedonism	1–6	4.43	1.18	3.55 (1.68)	4.38 (1.10)	4.52 (1.11)	4.52 (1.18)	L < LM M H^*^
5	Altruism	1–6	4.58	1.09	4.45 (1.54)	4.58 (1.04)	4.54 (1.05)	4.67 (1.11)	
6	Religion	1–4	1.75	1.03	1.82 (1.01)	1.70 (1.04)	1.78 (1.00)	1.74 (1.10)	
7	Spirituality	1–4	2.57	1.15	2.55 (1.14)	2.47 (1.16)	2.64 (1.12)	2.57 (1.22)	
**COGNITIVE**									
8	Mindful attention (aka acting w/ awareness)	1–6	4.42	0.96	3.42 (1.08)	3.96 (0.96)	4.44 (0.78)	5.18 (0.69)	L < LM < M < H^*^
9	Pausing before reacting (aka emotional reactiv ity)	1–6	4.14	1.18	3.50 (1.23)	3.95 (1.13)	4.07 (1.12)	4.63 (1.18)	L LM M < H^**^
10	Non-rumination	1–6	3.65	1.20	2.32 (0.85)	2.95 (0.96)	3.60 (1.04)	4.85 (0.75)	L < LM < M < H^*^
**AFFECTIVE**									
11	Positive affect	0–100	66.2	17.7	27.1 (13.7)	50.4 (9.81)	70.5 (9.00)	84.2 (8.82)	L < LM < M < H^***^
12	Life satisfaction	1–10	7.23	1.94	3.91 (2.39)	5.99 (1.90)	7.59 (1.37)	8.65 (1.03)	L < LM < M < H^***^
**PSYCHO-SOMATIC**									
13	Vitality	0–100	58.6	16.8	18.2 (11.0)	47.2 (10.6)	61.3 (9.86)	75.2 (9.47)	L < LM < M < H^***^
**SOCIAL**									
14	Positive relations with others	0–100	64.6	18.6	36.6 (18.3)	53.1 (16.2)	66.7 (14.9)	79.8 (12.0)	L < LM < M < H^***^

*SD, standard deviation; NB, multiple pair-wise comparisons with Tukey's test correction in ANOVA, with highest significant p value indicated*.

* *p < 0.05*,

** *p < 0.01*,

*** *p < 0.001*.

### Relationship between psycho-social resources and mental illness/suicidality

Table 3 shows that the 4-class index based on the level of psycho-social resources appears to distinguish the respondents robustly along mental illness and suicidality, not just recent morbidity corresponding to the mostly implicit time frame of the responses for psycho-social resources, but also in the past 12 months and even lifetime. Along all indicators, respondents with a low level of psycho-social resources demonstrate the worst level of recent health (e.g., 90.9% with serious mental illness in the past 4 weeks), but also in the past 12 months (e.g., 68.2% with major depression) and lifetime (e.g., nearly half reporting a suicide attempt). Although respondents with a high level of psycho-social resources demonstrate modest levels of current and 12-month mental morbidity, a quarter reported depression and suicidal ideation and 9.5% a suicide attempt in their lifetime.

**Table 3:** Overall health, mental illness, and suicidality by latent classes of psycho-social resources among gay men in Geneva, Switzerland, in 2011 (*n* = 428).

		**Psychosocial resources**				
**Family**	**Variable**	**Low (L) mean (SD) or %**	**Medium-low (LM) mean (SD) or %**	**Medium (M) mean (SD) or %**	**High (H) mean (SD) or %**	**Statistical significance**
**SUBJECTIVE GLOBAL HEALTH**						
	Subjective global health (1–5)	3.14 (1.17)	3.98 (0.61)	4.20 (0.69)	4.56 (0.52)	L < LM < M < H^***^
	Subjective global health (5 very good)	9.1	16.7	33.5	56.8	< 0.001
**SHORT-TERM DISABILITY**						
	Short-term disability due to physical health / illness < 2 wks	22.7	16.7	13.3	6.3	0.08
	Short-term disability due to mental health / illness < 2 wks	36.4	27.8	5.9	0.0	< 0.001
**NEGATIVE AFFECT**						
	Serious mental illness < 4 wks (0–24)	16.7 (3.34)	11.23 (3.33)	6.65 (2.71)	3.79 (2.74)	L > LM > M > H^***^
	Serious mental illness < 4 wks (>13)	90.9	32.4	1.5	1.1	< 0.001
	Major depression < 12 mon	68.2	26.9	4.9	2.1	< 0.001
	Self-reported depression < 12 mon	54.5	26.9	5.4	4.2	< 0.001
	Self-reported anxiety < 12 mon	50.0	25.9	5.4	0.0	< 0.001
	Self-reported depression lifetime	77.2	69.4	40.9	27.4	< 0.001
	Self-reported anxiety lifetime	81.8	50.9	27.1	9.5	< 0.001
**SUICIDALITY**						
	Suicidal ideation < 12 mon	72.7	32.4	8.9	2.1	< 0.001
	Suicide attempt < 12 mon	18.2	3.7	0.0	0.0	< 0.001
	Suicidal ideation lifetime	86.4	66.7	43.8	28.4	< 0.001
	Suicide attempt lifetime	45.5	28.7	9.4	9.5	< 0.001

SD, standard deviation. NB: multiple pair-wise comparisons with Tukey's test correction in ANOVA, with highest significant p value indicated - ^*^p < 0.05, ^**^p < 0.01,

*** *p < 0.001*.

In Table 4, we examine the relationship between psycho-social resources and recent mental illness outcomes more closely, by looking at psycho-social resources both in classes and singly, with and without controlling for select socio-demographic variables. Looking at a screening variable for serious mental illness in the past 4 weeks, we see that 10 psycho-social resources are significantly protective, yet 2 of them are significant risk factors at the bivariable level: internalized homophobia (OR = 1.82, 95% CI 1.38–2.39) and religion (OR = 1.32, 95% CI 1.03–1.69). The strongest protective factors at the bivariable level are purpose in life (OR = 0.39, 95% CI 0.28–0.55), non-rumination (OR = 0.40, 95% CI 0.30–0.53), and mindful attention (OR = 0.43, 95% CI 0.28–0.55).

**Table 4:** Recent mental illness outcomes by psycho-social resources among gay men in Geneva, Switzerland, in 2011 (*n* = 428).

**Family**	**Variable**	**Serious mental illness<4 wks (score >13)**				**Short-term disability due to mental health/illness<2 wks**			
		**OR**	**Model 1 AOR**	**Model 2 AOR**	**Model 3 AOR**	**OR**	**Model 1 AOR**	**Model 2 AOR**	**Model 3 AOR**
**PSYCHO-SOCIAL RESOURCES (INDIVIDUALLY)**									
**Core self-evaluation**									
1	Mastery	0.74 (0.67–0.81)^***^	1.05 (0.89–1.24)	1.05 (0.89–1.24)		0.87 (0.81–0.95)^**^	1.12 (0.99–1.27)	1.10 (0.97–1.25)	
2	Internalized homophobia (aka acceptance of one's homosexuality)	1.82 (1.38–2.39)^***^	1.17 (0.69–1.98)	1.17 (0.69–1.98)		1.35 (1.01–1.82)^*^	0.93 (0.62–1.38)	0.91 (0.61–1.35)	
**Conative**									
3	Purpose in life	0.39 (0.28–0.55)^***^	0.39 (0.21–0.73)^**^	0.39 (0.21–0.73)^**^	0.39 (0.22–0.71)^**^	0.64 (0.45–0.90)^*^	0.82 (0.49–1.35)	0.80 (0.48–1.32)	
4	Hedonism	0.80 (0.64–1.00)^*^	1.21 (0.80–1.82)	1.21 (0.80–1.82)		1.13 (0.87–1.46)	1.37 (0.99–1.90)	1.31 (0.94–1.83)	1.41 (1.04–1.92)^*^
5	Altruism	1.08 (0.84–1.39)	1.38 (0.89–2.13)	1.38 (0.89–2.13)		1.31 (0.98–1.75)	1.22 (0.87–1.70)	1.20 (0.86–1.67)	
6	Religion	1.32 (1.03–1.69)^*^	1.76 (1.03–2.98)^*^	1.76 (1.03–2.98)^*^	2.04 (1.30–3.18)^**^	1.20 (0.92–1.58)	1.21 (0.83–1.77)	1.28 (0.87–1.89)	1.31 (0.97–1.78)
7	Spirituality	1.16 (0.91–1.48)	1.34 (0.83–2.19)	1.34 (0.83–2.19)		1.15 (0.89–1.50)	1.12 (0.80–1.59)	1.13 (0.80–1.60)	
**Cognitive**									
8	Mindful attention (aka acting w/ awareness)	0.43 (0.32–0.57)^***^	0.78 (0.43–1.40)	0.78 (0.43–1.40)		0.51 (0.39–0.69)^***^	0.85 (0.56–1.28)	0.84 (0.56–1.28)	
9	Pausing before reacting (aka emotional reactivity)	0.71 (0.57–0.89)^**^	0.81 (0.53–1.23)	0.81 (0.53–1.23)		0.90 (0.71–1.14)	0.99 (0.73–1.34)	1.01 (0.74–1.38)	
10	Non-rumination	0.40 (0.30–0.53)^***^	1.16 (0.67–2.01)	1.16 (0.67–2.01)		0.45 (0.34–0.60)^***^	0.75 (0.50–1.14)	0.81 (0.53–1.22)	
**Affective**									
11	Positive affect	0.84 (0.81-0.88)^***^	0.83 (0.78–0.88)^***^	0.83 (0.78–0.88)^***^	0.84 (0.78–0.88)^***^	0.94 (0.92–0.95)^***^	0.94 (0.91–0.96)^***^	0.94 (0.91–0.97)^***^	0.93 (0.92–0.95)^***^
12	Life satisfaction	0.57 (0.49–0.66)^***^	0.96 (0.73–1.25)	0.96 (0.73–1.25)		0.69 (0.61–0.79)^***^	0.86 (0.68–1.09)	0.85 (0.67–1.08)	
**Psycho-somatic**									
13	Vitality	0.92 (0.90–0.94)^***^	0.99 (0.95–1.02)	0.99 (0.95–1.02)		0.95 (0.94–0.97)^***^	1.00 (0.97–1.02)	1.00 (0.97–1.02)	
**Social**									
14	Positive relations with others	0.94 (0.93–0.96)^***^	1.01 (0.97–1.04)	1.01 (0.97–1.04)		0.97 (0.96–0.98)^***^	1.01 (0.99–1.04)	1.01 (0.99–1.04)	
**PSYCHO-SOCIAL RESOURCES (LATENT CLASSES)**									
**4-class model**									
	Low		—	—			—	—	
	Medium-low		0.05 (0.01–0.22)^***^	0.05 (0.01–0.24)^***^			0.67 (0.26–1.77)	0.95 (0.35–2.56)	
	Medium		0.002 (0.000–0.01)^***^	0.002 (0.000–0.01)^***^			0.11 (0.04–0.31)^***^	0.14 (0.05–0.42)^***^	
	High		0.001 (0.000–0.01)^***^	0.001 (0.000–0.01)^***^			NA	NA	

* *p < 0.05*,

** *p < 0.01*,

*** *p < 0.001*.

*OR, odds ratio; AOR, adjusted odds ratios; NA, not available due to zero in the cells*.

*Model 1: 2 separate logistic regression models: a) all 14 psycho-social resources in one block and b) latent classes in one block*.

*Model 2: 2 separate logistic regression models controlling for socio-demographic covariates: a) all 14 psycho-social resources in one block and b) latent classes in one block*.

*Model 3: Most parsimonious logistic regression model controlling for socio-demographic covariates, starting with all 14 psycho-social resources*.

In Model 1 with the 4-class index (Nagelkerke *r*^2^ 0.54), respondents with higher levels of psycho-social resources are strongly protected against recent serious mental illness compared to those with low levels. In Model 1 with all 14 psycho-social resources as a block (Nagelkerke *r*^2^ 0.72), purpose in life (AOR = 0.39, 95% CI 0.21–0.73) and positive affect (AOR = 0.83, 95% CI 0.78–0.88) remain significantly protective at prior levels whereas religion becomes a stronger risk factor (AOR = 1.76, 95% CI 1.03–2.98). Controlling for socio-demographics in Model 2 does not change these findings; however, increasing age is independently protective (*p* = 0.04) with the 4-class index (Nagelkerke *r*^2^ 0.55) but not with the 14 psycho-social resources (Nagelkerke *r*^2^ 0.72). In Model 3 whereby variables are removed from Model 2 until the most parsimonious model is found (Nagelkerke *r*^2^ 0.71), the same three psycho-social resources from Models 1 and 2 remain significant, with increasing age remaining independently protective (*p* = 0.03).

As for the second outcome short-term disability due to mental/emotional problems in the past 2 weeks, 9 of the psycho-social resources are significantly associated with the outcome at the bivariable level, with all these resources being significantly protective except for internalized homophobia which is a significant risk factor (OR = 1.35, 95% CI 1.01–1.82). Among the significantly protective resources, mindful attention (OR = 0.51, 95% CI 0.39–0.69) and non-rumination (OR = 0.45, 95% CI 0.34–0.60) are once again the most strongly protective.

In Model 1 with the 4-class index (Nagelkerke *r*^2^ 0.26), respondents with medium and high levels of psycho-social resources are strongly protected against the outcome of short-term disability compared to those with low and medium-low levels (NB: no AOR generated for high category due to zero cases). In Model 1 with all 14 psycho-social resources as a block (Nagelkerke *r*^2^ 0.33), only positive affect remains significantly protective (AOR = 0.94, 95% CI 0.91–0.95), with most of the other resources moving toward parity. Controlling for socio-demographics in Model 2 does not change these findings, but increasing age is borderline protective (*p* = 0.07) with the 4-class index (Nagelkerke *r*^2^ 0.30) and independently protective (*p* = 0.002) with the block of 14 psycho-social resources (Nagelkerke *r*^2^ 0.33).

In Model 3 (Nagelkerke *r*^2^ 0.31), positive affect remains significantly protective (AOR = 0.93, 95% CI 0.92–0.95), with hedonism becoming a significant risk factor (AOR = 1.41, 95% CI 1.04–1.92) and religion a borderline significant risk factor (AOR = 1.31, 95% CI 0.97–1.78). Increasing age remains independently protective (*p* = 0.03). Since serious mental illness in the past 4 weeks itself may constitute a risk factor for short-term disability due to mental/emotional problems in the past 2 weeks, we added the former to Model 3 in a supplementary model (Nagelkerke *r*^2^ 0.32). Indeed, serious mental illness is a significant risk factor (AOR = 3.00, 95% CI 1.18–7.64), yet hedonism, positive affect, and age still remain independently significant at levels seen in Model 3.

### Psycho-social resources protective in stress-distress pathway

Table 5 examines the impact of a stressor—i.e., victimization—on three outcomes of distress —i.e., major depression, suicidal ideation, and suicide attempt in the past 12 months—and explores potential mediation by psycho-social resources using a similar approach as in **Table 4**. At the bivariable level, victimization is a significant risk factor for major depression (OR = 2.24, 95% CI 1.26–4.00). However, entering the 4-class index into the model shows that higher levels of psycho-social resources are independently and strongly protective and pushes the AOR for victimization toward parity, thereby losing statistical significance (AOR = 1.79, 95% CI 0.92–3.47; Nagelkerke *r*^2^ 0.33), similarly after adjusting for socio-demographics (AOR = 1.59, 95% CI 0.81–3.14; Nagelkerke *r*^2^ 0.35). Looking at potential mediation by psycho-social resources singly in simple logistic regression models, non-rumination (AOR = 0.43, 95% CI 0.32–0.57), positive affect (AOR = 0.93, 95% CI 0.91–0.94), vitality (AOR = 0.94, 95% CI 0.92–0.95), and positive relations with others (AOR = 0.95, 95% CI 0.94–0.97) are each independently protective and decrease the AOR for victimization below statistical significance.

**Table 5:** Major depression and suicidality by victimization in the past 12 months and current psycho-social resources among gay men in Geneva, Switzerland, in 2011 (*n* = 428).

**Family**	**Variable**	**Major depression<12 months**				**Suicidal ideation<12 months**				**Suicide attempt<12 months**			
		**(A)OR**	**Model 1 AOR**	**Model 2 AOR**	**Model 3 AOR**	**(A)OR**	**Model 1 AOR**	**Model 2 AOR**	**Model 3 AOR**	**(A)OR**	**Model 1 AOR**	**Model 2 AOR**	**Model 3 AOR**
**PSYCHO–SOCIAL RESOURCES (INDIVIDUALLY)**													
**Core self-evaluation**													
1	Mastery	0.82 (0.75–0.89)^***^	1.14 (1.00–1.30)^*^	1.14 (1.00–1.30)^*^		0.77 (0.71–1.99)^***^	1.04 (0.93–1.16)	1.05 (0.93–1.17)		0.66 (0.54–0.81)^***^	0.69 (0.45–1.07)	0.67 (0.42–1.07)	0.66 (0.51–0.86)^**^
2	Internalized homophobia (aka acceptance of one's homosexuality)	1.45 (1.09–1.94)^*^	0.99 (0.66–1.48)	0.99 (0.66–1.48)		1.43 (1.10–1.86)^**^	1.01 (0.70–1.47)	1.00 (0.69–1.46)		2.31 (1.24–4.29)^**^	4.41 (1.14–17.0)^*^	4.47 (1.01–19.9)^*^	2.73 (1.12–6.64)^*^
**Conative**													
3	Purpose in life	0.56 (0.40–0.78)^**^	0.99 (0.61–1.62)	0.99 (0.61–1.62)		0.50 (0.33–0.63)^***^	0.86 (0.56–1.32)	0.89 (0.58–1.36)		0.56 (0.26–1.20)	5.59 (1.06–29.4)^*^	3.88 (0.71–21.2)	
4	Hedonism	0.87 (0.69–1.10)	1.15 (0.84–1.58)	1.15 (0.84–1.58)		0.88 (0.72–1.09)	1.14 (0.86–1.52)	1.15 (0.87–1.53)		0.67 (0.38–1.16)	1.70 (0.65–4.46)	1.43 (0.54–3.78)	
5	Altruism	0.98 (0.75–1.27)	0.99 (0.71–1.38)	0.99 (0.71–1.38)		0.83 (0.66–1.04)	0.80 (0.59–1.08)	0.79 (0.59–1.08)		0.76 (0.41–1.42)	0.84 (0.34–2.05)	0.84 (0.32–2.22)	
6	Religion	0.90 (0.68–1.20)	0.84 (0.55–1.28)	0.84 (0.55–1.28)		0.77 (0.58–1.02)	0.69 (0.47–1.03)	0.69 (0.46–1.04)	0.69 (0.49–0.98)^*^	0.46 (0.14–1.50)	0.18 (0.03–1.23)	0.23 (0.03–1.80)	
7	Spirituality	0.96 (0.75–1.23)	0.98 (0.70–1.37)	0.98 (0.70–1.37)		0.96 (0.77–1.20)	1.07 (0.79–1.44)	1.04 (0.77–1.42)		0.61 (0.32–1.19)	0.33 (0.09–1.20)	0.27 (0.06–1.22)	0.39 (0.15–0.98)^*^
**Cognitive**													
8	Mindful attention (aka acting w/ awareness)	0.39 (0.28–0.54)^***^	0.61 (0.40–0.94)^*^	0.61 (0.40–0.94)^*^	0.59 (0.41–0.84)^**^	0.40 (0.30–0.54)^***^	0.69 (0.46–1.01)	0.68 (0.46–1.01)	0.70 (0.48–1.01)	0.43 (0.23–0.79)^**^	1.20 (0.33–4.42)	1.38 (0.36–5.24)	
9	Pausing before reacting (aka emotional reactiv ity)	0.72 (0.57–0.91)^**^	0.81 (0.59–1.12)	0.81 (0.59–1.12)		0.68 (0.55–0.84)^***^	0.74 (0.56–0.99)^*^	0.72 (0.54–0.97)^*^	0.72 (0.55–0.95)^*^	0.75 (0.43–1.31)	0.87 (0.42–1.81)	0.95 (0.44–2.03)	
10	Non-rumination	0.43 (0.32–0.57)^***^	0.73 (0.47–1.13)	0.73 (0.47–1.13)		0.41 (0.31–0.54)^***^	0.66 (0.45–0.95)^*^	0.63 (0.43–0.92)"	0.65 (0.46–0.92)^*^	0.36 (0.17–0.75)^**^	1.04 (0.28–3.95)	1.26 (0.33–4.82)	
**Affective**													
11	Positive affect	0.93 (0.91–0.94)^***^	0.95 (0.92–0.98)^**^	0.95 (0.92–0.98)^**^	0.95 (0.93–0.98)^***^	0.94 (0.92–0.95)^***^	0.96 (0.94–0.99)^**^	0.96 (0.93–0.99)^**^	0.96 (0.94–0.98)^***^	0.92 (0.89–0.96)^***^	0.90 (0.82–0.99)^*^	0.93 (0.85–1.02)	
12	Life satisfaction	0.65 (0.56–0.75)^***^	0.98 (0.77–1.24)	0.98 (0.77–1.24)		0.61 (0.54–0.70)^***^	0.85 (0.70–1.05)	0.86 (0.70–1.05)	0.82 (0.69–0.97)^*^	0.59 (0.44–0.78)^***^	0.88 (0.47–1.67)	0.79 (0.36–5.24)	
**Psycho–somatic**													
13	Vitality	0.94 (0.92–0.95)^***^	0.97 (0.95–1.00)^*^	0.97 (0.95–1.00)^*^	0.97 (0.95–1.00)^*^	0.95 (0.94–0.97)^***^	1.00 (0.98–1.02)	1.00 (0.97–1.02)		0.95 (0.92–0.98)^**^	1.05 (0.98–1.12)	1.04 (0.97–1.12)	
**Social**													
14	Positive relations with others	0.95 (0.94–0.97)^***^	0.98 (0.96–1.01)	0.98 (0.96–1.01)		0.95 (0.94–0.97)^***^	0.99 (0.96–1.01)	0.99 (0.96–1.01)		0.94 (0.91–0.98)^**^	0.99 (0.92–1.06)	0.99 (0.91–1.08)	
													
**PSYCHO–SOCIAL RESOURCES (LATENT CLASSES)**													
**4-class model**													
	Low		—	—			—	—			—	—	
	Medium–low		0.17 (0.06–0.45)^***^	0.21 (0.08–0.58)^**^			0.18 (0.07–0.50)^**^	0.19 (0.07–0.56)^**^			0.16 (0.04–0.73)^*^	0.25 (0.05–1.16)	
	Medium		0.03 (0.008–0.08)^***^	0.03 (0.01–0.09)^***^			0.04 (0.01–0.11)^***^	0.04 (0.01–0.11)^***^			NA	NA	
	High		0.01 (0.002–0.06)^***^	0.01 (0.003–0.08)^***^			0.008 (0.001–0.04)^***^	0.009 (0.002–0.05)^***^			NA	NA	
**STRESSOR**													
**Victimization<12 months**													
	bivariate	2.24 (1.26–4.00)^**^				1.41 (0.82–2.43)				4.58 (1.08–19.5)^*^			
	multivariate with psycho–social resources individually		1.61 (0.78–3.33)	1.61 (0.78–3.33)	1.54 (0.76–3.13)		0.92 (0.46–1.83)	1.05 (0.52–2.12)	1.05 (0.53–2.07)		2.27 (0.27–19.3)	1.13 (0.09–14.5)	0.72 (0.10–5.32)
	multivariate with psycho–social resources in latent classes		1.79 (0.92–3.47)	1.59 (0.81–3.14)			1.04 (0.56–1.94)	1.04 (0.56–1.94)			3.31 (0.72–15.3)	2.14 (0.44–10.4)	

* *p < 0.05*,

** *p < 0.01*,

*** *p < 0.001*.

*OR, odds ratio; AOR, adjusted odds ratios; NA, not available due to zero in the cells*.

*Model 1: 2 Separate logistic regression models with victimization: (a) All 14 psycho-social resources in one block and (b) latent classes in one block*.

*Model 2: 2 Separate logistic regression models with victimization controlling for socio-demographic covariates: (a) All 14 psycho-social resources in one block and (b) latent classes in one block*.

*Model 3: Most parsimonious logistic regression model with victimization controlling for socio-demographic covariates, starting with all 14 psycho-social resources*.

In Model 1 with all 14 psycho-social resources as a block (Nagelkerke *r*^2^ 0.42), mindful attention (AOR = 0.61, 95% CI 0.40–0.94), positive affect (AOR = 0.95, 95% CI 0.92–0.98), and vitality (AOR = 0.97, 95% CI 0.95–1.00) remain independently protective factors, with mastery (AOR = 1.14, 95% CI 1.00–1.30) flipping from being a protective to an independent risk factor. However, just as in the analyses with the 4-class index, the AOR for victimization decreases and loses statistical significance (AOR = 1.61, 95% CI 0.78–3.33). Controlling for socio-demographics in Model 2 does not change these findings one bit (Nagelkerke *r*^2^ 0.42). In Model 3 (Nagelkerke *r*^2^ 0.40), mindful attention (AOR = 0.59, 95% CI 0.41–0.84), positive affect (AOR = 0.95, 95% CI 0.93–0.98), and vitality (AOR = 0.97, 95% CI 0.95–1.00) remain independently protective factors which decrease the impact of victimization (AOR = 1.54, 95% CI 0.76–3.13) on major depression in the past 12 months below statistical significance.

Victimization is not a significant risk factor for suicidal ideation in the past 12 months at the bivariable level (OR = 1.41, 95% CI 0.82–2.43), and adding psycho-social resources—which are significantly protective factors in both 4-class index and single permutations—to the models just moves the AOR for victimization to parity. In Model 3 (Nagelkerke *r*^2^ 0.40), religion (AOR = 0.69, 95% CI 0.49–0.98), mindful attention (AOR = 0.70, 95% CI 0.48–1.01), pausing before acting (AOR = 0.72, 95% CI 0.55–0.95), non-rumination (AOR = 0.65, 95% CI 0.46–0.92), positive affect (AOR = 0.96, 95% CI 0.94–0.98), and life satisfaction (AOR = 0.82, 95% CI 0.69–0.97) all protect independently from suicidal ideation in the past 12 months, as does increasing age (*p* = 0.04).

Victimization is a strong risk factor for suicide attempt in the past 12 months at the bivariable level (OR = 4.58, 95% CI 1.08–19.5). Introducing the 4-class index into the model, higher levels of psycho-social resources remain independently and strongly protective (NB: no AOR generated for medium and high categories due to zero cases) and decrease the AOR for victimization below statistical significance (AOR = 3.31, 95% CI 0.72–15.3; Nagelkerke *r*^2^ 0.36). Adjusting for age pushes the AOR for victimization even lower and increases the variance explained (AOR = 2.14, 95% CI 0.44–10.4; Nagelkerke *r*^2^ 0.44). Looking at the mediation effect of psycho-social resources singly, the 8 psycho-social resources which are significantly protective for suicide attempt each push victimization toward parity, thereby losing statistical significance.

In Model 1 with all 14 psycho-social resources as a block (Nagelkerke *r*^2^ 0.56), the AOR for victimization drops to 2.27 (95% CI 0.27–19.3), with positive affect remaining a protective factor (AOR = 0.90, 95% CI 0.82–0.99) and internalized homophobia (AOR = 4.41, 95% CI 1.14–17.0) and purpose in life (AOR = 5.59, 95% CI 1.06–29.4) as strong risk factors. Adjusting for age in Model 2 (Nagelkerke *r*^2^ 0.61), victimization drops to near parity (AOR = 1.13, 95% CI 0.09–14.5) with only internalized homophobia (AOR = 4.47, 95% CI 1.01–19.9) remaining as a significant risk factor. In the most parsimonious Model 3 (Nagelkerke *r*^2^ 0.51), internalized homophobia (AOR = 2.73, 95% CI 1.12–6.64) remains a significant risk factor at bivariable levels, but mastery (AOR = 0.66, 95% CI 0.51–0.86) and spirituality (AOR = 0.39, 95% 0.15–0.98) become independently protective factors, together with increasing age (*p* = 0.01).

## Discussion

This relatively elaborate profile of psycho-social resources presents a more complete picture of gay men's mental health than has been available to date based on psychiatric symptoms and morbidity alone. Using their own scores, men were grouped into meaningful classes with (relatively) low, medium, or high levels of psycho-social resources. This 4-class index of current/recent psycho-social resources appears to be remarkably robust in distinguishing the gay male sample along multiple mental illness and suicidality outcomes in the past 2–4 weeks, in the past 12 months, and lifetime, with logistic regression models underscoring the association between psycho-social resources and mental morbidity (1). Of note, psycho-social resources appear to buffer the effects of serious stressors such as victimization in leading to distressful outcomes such as major depression and suicide attempt in the past 12 months.

### Psycho-social resources associated with mental illness and suicidality

Individually, most psycho-social resources are consistently protective against multiple mental illness and suicidality outcomes in bivariable analyses. The dramatic reduction in the number of independently protective psycho-social resources when moving from bivariable to multivariable analyses suggests possible inter-relationships which require closer examination. While psycho-social resources explain a considerable proportion of variance in the logistic regression models, the percentage varies considerably depending on the outcome. By examining multiple psycho-social resources and multiple mental morbidity outcomes, distinctive results for each of the five outcomes of mental illness and suicidality belie simplistic generalizations.

Positive affect as measured by the MHI-5 is a consistent, independent protective factor for four of the five outcomes, and since it is also the indicator closest to those for mental illness, its relationship to other psycho-social resources may be a good place to start examining inter-relationships. The importance of positive affect in the models with mental morbidity outcomes is supported by a large body of evidence that mood and anxiety disorders are characterized in part by deficiencies in positive affect (54) and that positive affect may protect against mental illness directly via physiological systems but also indirectly via improved cognitive resources (1, 55).

The two cognitive resources related to mindfulness—attention and non-rumination—are consistently the most strongly protective factors across all outcomes and remain independently protective for major depression and suicidal ideation in the past 12 months. In the literature, dispositional mindfulness has been shown to mediate the relationship between neuroticism and depressive symptoms / psychological distress (21–23) which may be particularly relevant given the role of neuroticism in mental illness and suicidality among sexual minority men (19). However, it is unclear why the independent effects do not apply to recent non-specific mental morbidity outcomes. Although higher mindfulness was associated with lower psychological distress in the past 4 weeks, a longitudinal study among gay men aged 40 years and over showed that dispositional mindfulness did not predict psychological distress at 12-month follow-up (27).

Religion is peculiar, since it is one of several conative resources which are insignificant at the bivariable level for most outcomes, yet functions as an independent risk factor for recent mental morbidity but a protective factor for suicidal ideation in the past 12 months. In the general literature, religion is protective against both depression and suicide attempts—with inconsistent evidence for suicidal ideation (56)—constituting a risk factor in only a small number of studies (57). The evidence suggests that religion is similarly protective against depression and suicidality among adolescents (58); however, among sexual minority adolescents, religion has null association with depression in most studies (59), except in the presence of negative experiences or conflict when religion becomes a risk factor among all adolescents, including sexual minorities. In two recent studies, religion was protective against suicidal ideation (60) and suicide attempts (61) among heterosexual adolescents and young adults but not their sexual minority counterparts and actually acted as a risk factor for suicide attempts amongst the latter. Indeed, religious struggle constitutes a clear risk factor for mental illness generally (62), and qualitative research has shown that due to stigmatization of homosexuality, sexual minorities may experience religious conflict more often, resulting in shame/guilt, depression, and suicidal ideation (63, 64).

While positive relations with others are associated with other psycho-social resources and all mental illness and suicidality outcomes at the bivariable level, it is not an independently protective factor in any multivariable model, a finding which has also been evidenced elsewhere (65). Generally, social support has been shown to be protective against depression among adolescents and adults (66). Although social support has been linked to the psycho-social resources of self-esteem and self-acceptance (including internalized homophobia) among sexual minority adolescents (67, 68) and adults (69), its link to depression, anxiety, and suicidal ideation is equivocal among adolescents (59, 67). While social support was associated with psychological distress cross-sectionally, it did not predict psychological distress longitudinally in cohorts of young LGBTQ (70) and gay men aged 40 years and over (27). Although positive relations with others appear to have no independent effects on mental morbidity outcomes in this sample, they may exert indirect effects via other psycho-social resources (8, 67, 71) as have been found for parental social support in studies among adolescents generally (66).

### Psycho-social resources protective in stress-distress pathway

Much of the evidence linking vicitimization with mental morbidity comes from studies among adolescents. Meta-analyses have confirmed that victimization leads to higher risk of poor mental health, depression, anxiety, suicidal ideation, and suicide attempt among adolescents, with pooled OR ranging from 1.60 to 2.55 [(72, 73)]. Our findings confirm higher levels of depressive symptoms yet mixed findings for suicidality among sexual minority adolescents (11).

Cross-sectional studies among general population and sexual minority adults and adolescents have demonstrated the protective role of several psycho-social resources in the victimization-distress pathway. In a national sample of US adults, sense of control (i.e., mastery and perceived constraints), but not social support, moderated the impact of childhood physical abuse on negative affect in the past month and self-reported global health (74). In a community sample of gay men in the US, several forms of victimization in the past 2 years remained independently associated with depressive symptoms in the past year, alongside most of the psycho-social resources included in the model—i.e., self-esteem (the strongest predictor), internalized homophobia, and social support (with some forms being protective whilst others being risk factors) (75). Among sexual minority youths in the US, self-acceptance (i.e., self-esteem and comfort with one's sexual orientation) and social support from family (i.e., acceptance, protection, and relations) mediated the impact of victimization on psychiatric symptoms in the past week in one study (76), yet neither social support from family nor from friends mediated the impact of lifetime victimization on psychiatric symptoms in the past week in another (77).

Higher levels of mindfulness protected against the effect of victimization due to sexual orientation but not due to ethnicity on depressive symptoms in the past week among young Latino sexual minority youth in the US (28). Mindfulness has also been shown to protect against other stressors. Mindfulness and positive affect were independently protective factors in attenuating the impact of discrimination (racism) in the past 12 months on recent depressive symptoms (78) and anxious arousal symptoms (79) in the US, and mindfulness attenuated the impact of sexuality- and age-based discrimination in the past 2 years on recent psychological distress in an Australian sample of gay men aged 40 and over (26). Mindfulness—especially the facet acting with awareness—moderated the impact of perceived stress in the past month on current depression, but not current anxiety in the Swedish general population (24).

### Fewer psycho-social resources among gay men

Population norms are not available for many psycho-social resources in this paper, nor do most of their scores have clear cut-offs or categories, rendering interpretation challenging. As in this study, scores for psycho-social resources tend to be used relatively (higher vs. lower) within a study population, but this state of affairs is not entirely satisfactory when studying vulnerable populations whose scores may differ significantly from those in the referent general population. As this study design yields a sample that is representative of all gay men who fall within the sampling scheme of meeting points (33, 34) and by extension gay men who live in that corresponding urban area (80), we compare our study means with available population means to identify possible disparities in psycho-social resources.

Using data matched for sex, age, region, and nationality between the 2002 Geneva Gay Men's Health Survey and the 2002 Swiss Health Survey, more gay men demonstrated low mastery than matched men from the general population (53.9% vs. 31.1%, *p* < 0.00001), even though a quarter of men in both samples had high mastery. Gay men in 2011 had significantly lower altruism scores (vs. mean 4.97, *SD* 0.82, *p* < 0.0001), marginally higher hedonism scores (vs. mean 4.30, *SD* 1.23, *p* = 0.08), and significantly lower life satisfaction scores (vs. mean 8.23, *SD* 1.65, *p* < 0.0001) than general population men in the Swiss arm of the 2012 European Social Survey. The mean scores for positive affect and vitality are comparable to population means from the neighboring canton of Vaud (81), yet significantly lower than those from a young to mid-age university-based sample from Geneva (82) with a more similar socio-demographic profile.

Even more robust evidence of disparities in psycho-social resources come from large population-based surveys elsewhere that include sexual orientation. Among US adults aged 25–74 years, men and women identifying as gay, lesbian, or bisexual had significantly lower scores for self-acceptance, environmental mastery, purpose in life, and positive relations with others than their heterosexual counterparts, with no differences for autonomy and personal growth (83). Findings from the US General Social Survey showed stronger altruistic values among homosexually experienced men than their heterosexual counterparts, yet empathic concern and actual altruistic and reciprocal behaviors did not differ by sexual orientation (84). National data have evidenced significantly lower levels of life satisfaction among sexual minority adults in Australia and the UK (85) and same-sex attracted adolescents in the Netherlands (86) and Iceland, where there was also lower social support from both friends and family (87). In fact, for many of the supportive resources commonly linked to positive outcomes among adolescents, the evidence suggests fewer are available to sexual minority adolescents than their heterosexual peers (88), pointing to early onset of psycho-social disparities.

### Young gay men with highest stress, highest distress, and fewest psycho-social resources

Increasing age was independently protective for all mental morbidity outcomes except major depression in the past 12 months—i.e., young gay men under 25 years were at highest risk. Data from Switzerland have established higher risk of mental illness and suicidality among gay men generally, with 12-month prevalences highest among young gay men (18, 19). Data from the 2002 Geneva Gay Men's Health Survey also showed that gay men were 3–4 times more likely to have been a victim of violence in the past 12 months (34% in 2002 and 27% in 2011) than their matched counterparts in the general population (53), with the highest 12-month prevalences among gay men under 25 years. A higher risk of victimization was also found among sexual minority mid-adolescents in a 2014 school-based survey in the cantons of Vaud and Zurich (89). In summary, the highest levels of suicidality and victimization are found among gay men under 25 years which are commensurate with age trends in the general population internationally (10, 90, 91).

In a large convenience sample in the US, age was an independent mediator in the relationship between victimization and depressive symptoms among gay men, but not among lesbians (75). The age-sensitivity in prevalences of both stressor and distress was most clearly evidenced in a cohort of LGBTQ youth in the US, whereby both victimization in the past 6 months and psychological distress in the past week decreased over time as probands progressed from late adolescence into young adulthood (70). In fact, a meta-analysis found age as the only significant moderator between bullying victimization and mental health problems and suicidality among sexual minority youth (12).

The present findings have shown that young gay men have the lowest level of psycho-social resources overall and specifically of cognitive resources such as mindful attention and non-rumination but also of positive affect. Review articles have not elucidated age/developmental trends in psycho-social resources (1, 4), but there are some indications from population-based studies. The Canadian NPHS confirmed highest AOR of depression and distress in the youngest age groups but also an upside-down U curve for both mastery and self-esteem, peaking in the 40–49 age group (31). Although a population-based study in Sweden found increasing mindfulness scores for select facets with increasing age, higher education, and higher income (24), our findings in this gay male sample replicate lower scores for mindful awareness and non-rumination only among gay men under 25 years.

### Limitations

This cross-sectional study does not permit assessments of causality or temporality in the associations presented. This limitation is particularly relevant for the examination of psycho-social resources as potential buffers in the stress-distress pathway. Although we chose a serious stressor—i.e., vicitimization—and serious indicators of distress—i.e., major depression and suicidality—with the same time frame—i.e., in the past 12 months—we cannot preclude instances where the distress might have preceded the stressor. The temporality of psycho-social resources is a general issue, since they are usually measured without explicit reference to any time frame. Although the literature supports a dispositional quality for many of the psycho-social resources—even positive affect (92)—studies also suggest fluctuation across time and the life course for some (31). For example, although the mean score for internalized homophobia did not change between 2002 and 2011, it may differ currently. Good practice dictates that just as the internal consistency of these scales needs to be re-assessed for every sample, the same applies to latent classes which may change between samples or across time.

There is an issue of valence for some of the indicators used in this paper, but this is another general issue for the field of psycho-social resources as a whole. Although psycho-social resources are positive in valence (1), some are measured using positive items, some using negative items, and some using a mix of positive and negative items. For example, the general concept of self-acceptance was measured by an indicator of self-acceptance of one's homosexuality—a key factor according to prior qualitative research in this population (93, 94)—which was operationalized in part by a purely negative indicator of internalized homophobia designed to capture ego-dystonic homosexuality (36). Both gay-specific and generalized indicators of self-acceptance should be considered in future work among gay men and include masculinity/femininity. However, findings among LGB youths suggest that valence matters: although internalized homophobia was consistently associated with depression and drug use, positive permutations of self-acceptance of one's homosexuality were not related to depression or anxiety (67).

Finally, the relatively large number of psycho-social resources measured using ordinal/continuous scores created some issues with both latent class analyses and logistic regression models. For the latter, we decided to forego the examination of interaction terms between individual psycho-social resources due to the large number of potential combinations. This is an important limitation, since many papers using models with just a handful of psycho-social resources identify significant interactions between them. The loss of fine-grained analyses is offset in part by the presentation of a more elaborate profile of psycho-social resources juxtaposed against multiple mental morbidity outcomes. The larger number of psycho-social resources also precluded the use of structural equation modeling which requires models whereby the inter-relationships between the individual psycho-social resources—treated as a single layer in this paper—are mapped out a priori, yet the current state of the art does not facilitate the construction of such an elaborate model. The analyses presented in this paper can inform subsequent model building.

### Conclusions

There is considerable evidence of health disparities among gay men characterized by higher levels of stress and distress. Psycho-social resources are distributed unequally in populations (1, 3, 9), and evidence of disparities in several psycho-social resources among gay men has been presented. Psycho-social resources have been used to explain established health disparities among several groups (4), and this paper has provided additional evidence that higher levels of psycho-social resources are strongly and negatively associated with mental illness and suicidality among gay men.

Both collectively and individually, psycho-social resources protect against the impact of a severe stressor such as victimization on indicators of distress—i.e., major depression and suicidality—in the past 12 months. But given the strength of these associations in the presence and absence of a stressor, psycho-social resources should not only be considered protective factors but also compensatory/promotive factors (95). As such, strategies to promote psycho-social resources may not only prevent or alleviate mental illness but may also promote positive mental health. Of note, several types of psycho-social resources are relevant, as resources in five different families remain significant in multivariable models. Since the individual psycho-social resources in the multivariable models differ depending on the mental illness outcome and possibly also time period and since some psycho-social resources may exert indirect effects via other psycho-social resources, efforts targeting overall mental health may need to promote multiple psycho-social resources.

Due to the unfortunate confluence of highest stress, highest distress, and fewest psycho-social resources among young gay men, these adolescents and young adults should be prioritized in future studies and interventions on multiple psycho-social resources. To date, only a handful of group-level interventions targeting sexual minority youth have been evaluated scientifically in North America, but results appear promising (96–98). The large body of research and interventions promoting socio-emotional competencies among children and adolescents (99) and mindfulness-based interventions (MBI) targeting adolescents and adults (100–102) may be particularly informative. Although increasingly offered to sexual minorities, we are aware of only two published studies among adults in North America: Mindfulness-based Stress Reduction (MBSR) improved positive affect and mindfulness and decreased depression in a randomized controlled trial (RCT) among HIV-positive gay men (103), and Acceptance and Commitment Therapy (ACT) improved internalized homophobia, mindfulness, and social support and decreased stress, depression, and anxiety in a small group of gay men and lesbians (104).

## Ethics statement

Health survey data collected anonymously do not require approval by cantonal ethics committees in Switzerland. Potential respondents were informed both orally and in writing about the general subject matter of the survey and the anonymous, yet secure, data collection and handling procedures.

## Author contributions

JW and MH conceived and directed the project. JW and A-EA conceived the article. JW performed the data analysis and drafted the article. All authors contributed to interpretation of the findings as well as the development of the manuscript.

### Conflict of interest statement

The authors declare that the research was conducted in the absence of any commercial or financial relationships that could be construed as a potential conflict of interest.
